# The Use of Thrombopoietin Receptor Agonists for Correction of Thrombocytopenia prior to Elective Procedures in Chronic Liver Diseases: Review of Current Evidence

**DOI:** 10.1155/2016/1802932

**Published:** 2016-10-09

**Authors:** Kamran Qureshi, Shyam Patel, Andrew Meillier

**Affiliations:** ^1^Department of Medicine, Section of Gastroenterology, Temple University Lewis Katz School of Medicine, Philadelphia, PA, USA; ^2^Department of Medicine, Internal Medicine Residency Program, Temple University Hospital, Philadelphia, PA, USA

## Abstract

Patients with chronic liver diseases (CLD) undergo a range of invasive procedures during their clinical lifetime. Various hemostatic abnormalities are frequently identified during the periprocedural work-up; including thrombocytopenia. Thrombocytopenia of cirrhosis is multifactorial in origin, and decreased activity of thrombopoietin has been identified to be a major cause. Liver is an important site of thrombopoietin production and its levels are decreased in patients with cirrhosis. Severe thrombocytopenia (platelet counts < 60–75,000/*µ*L) is associated with increased risk of bleeding with invasive procedures. In recent years, compounds with thrombopoietin receptor agonist activity have been studied as therapeutic options to raise platelet counts in CLD. We reviewed the use of Eltrombopag, Romiplostim, and Avatrombopag prior to various invasive procedures in patients with CLD. These agents seem promising in raising platelet counts before elective procedures resulting in reduction in platelet transfusions, and they also enabled more patients to undergo the procedures. However, these studies were not primarily aimed at comparing bleeding episodes among groups. Use of these agents had some adverse consequences, importantly being the occurrence of portal vein thrombosis. This review highlights the need of further studies to identify reliable methods of safely reducing the provoked bleeding risk linked to thrombocytopenia in CLD.

## 1. Introduction

The prevalence of thrombocytopenia in chronic liver disease (CLD) is 6% and is noticed to be as high as 70% in patients with liver cirrhosis [1]. The level of thrombocytopenia is associated with the severity of liver disease and the degree of portal hypertension [2]. Multiple theories are proposed to suggest the etiology of thrombocytopenia in CLD. It is believed that there is increased sequestration of platelets in the spleen that occurs as a consequence of splenomegaly [3], which is oftentimes seen in patients with cirrhosis and portal hypertension. The toxic effects of alcohol [4], viral-induced thrombocytopathy [5], and bone marrow suppression are also known to cause thrombocytopenia. The treatment of Hepatitis B Virus (HBV) or Hepatitis C Virus (HCV) infection with interferon causes bone marrow suppression and, subsequently, thrombocytopenia in such patients with CLD [6]. Autoantibodies have been identified in HCV infection and it is thought that these antibodies enhance the removal of platelets by the splenic and hepatic reticuloendothelial system [7]. Thrombopoietin (TPO), also known as Megakaryocyte Growth and Development Factor (MGDF) or c-MpL ligand, is a hormone which is synthesized in the liver and dominantly regulates the process of megakaryocytopoiesis [8]. TPO acts on c-MpL receptor on the surface of megakaryocytes and stimulates various steps of platelet production within the bone marrow [9]. TPO generation in turn is regulated by the rate of platelet cycling (production and destruction), as well as the synthetic function of liver [10]. Several studies have argued about the relative majority influence on this multifactorial etiology of thrombocytopenia in CLD [11].

Platelets play a critical role in hemostasis; however, thrombocytopenia in cirrhosis does not significantly increase the risk of spontaneous bleeding [12]. Though the number of circulating platelets is reduced, there is an increased expression of endothelium-derived von Willebrand factor, which makes the platelets more adherent to the endothelium and maintains the primary hemostasis at the site of bleeding [13]. Platelets also influence secondary hemostasis by participating in thrombin generation alongside the coagulation system. In in vitro studies, thrombin generation was found to be within the normal range despite mild to moderate thrombocytopenia in liver cirrhosis [14]. The threshold of 60,000/*μ*L platelet count was able to secure values of thrombin generation equal to the lower limit of the normal range [14]. In vivo evidence came from the post hoc analysis of the Hepatitis C Antiviral Long-term Treatment against Cirrhosis (HALT-C) trial. The complication rates in the 2740 liver biopsies that were performed in patients with bridging fibrosis and cirrhosis due to chronic HCV infection were assessed [15]. There were only 16 cases reported with the complication of bleeding; the bleeding rate was higher among patients with platelet counts less than 60,000/*μ*L [15]. This observation has led to a general acceptance that the platelet count above 50–60,000/*μ*L range is considered safe before most invasive procedures in patients with liver cirrhosis.

Management of periprocedural bleeding risk is a common clinical scenario in CLD. CLD patients frequently require invasive diagnostic and therapeutic procedures, such as liver biopsies, variceal band ligation, or percutaneous procedures for hepatocellular carcinoma (HCC). Traditionally, the treatment options for thrombocytopenia in CLD have been platelet transfusions, splenic artery embolization, splenectomy, and Transjugular Intrahepatic Portosystemic Stent (TIPS) placement [16–19]. Since its identification, TPO and its receptor have been pursued for the development of pharmacological agents to correct thrombocytopenia of CLD. TPO receptor agonists appear to be a good choice and have been routinely used in the treatment of chronic Idiopathic Thrombocytopenic Purpura (ITP), but their use in the treatment of thrombocytopenia prior to invasive procedures in CLD patients is relatively recent [20].

This paper reviews the only 4 clinical studies reported so far that have evaluated TPO receptor agonists for the correction of thrombocytopenia prior to elective procedures in CLD patients.  Table 1 summarizes these studies details and outcomes.

## 2. Rationale of Use of TPO Agonists in CLD

In a study done by Koruk et al. in 2002 [21], it was shown that serum TPO levels decreased as the degree of cirrhosis progressed. This study included 18 patients with chronic hepatitis infection, 48 with liver cirrhosis, and 27 control patients with no liver disease. Serum TPO levels were measured in each of these three cohorts. The mean serum TPO level in the chronic hepatitis group was 100.9 pg/mL, which was very similar to the mean TPO level in the control group (97.6 pg/mL). However, the mean TPO level in cirrhotic group was found to be 69.6 pg/mL, which was significantly lower when compared to the other two groups. In addition, as the degree of cirrhosis progressed (measured by the Child-Pugh score), the level of serum TPO was lower. Thrombocytopenia was seen more commonly in the cirrhosis group (65%). It was thus concluded that impaired production of TPO may contribute to the development of thrombocytopenia in advanced stage liver disease [21].

These findings led to the investigation of agents that act on the TPO receptor in order to correct thrombocytopenia in various clinical situations. Among the recombinant TPO agonists which have been developed for the use in humans, Eltrombopag, Romiplostim (first generation), and Avatrombopag (first and second generation) have shown to increase platelet production in humans. These agents act via c-MpL ligand mediated activation of the JAK-STAT and MAP kinase pathways [22, 23].

## 3. Experience of Use of TPO Agonists in CLD Patients

Eltrombopag is a small nonpeptide molecule that is taken orally, leads to a sustained increase in the platelet count by interacting with the transmembrane domain of the human TPO receptor, and initiates signaling cascades that induce proliferation and differentiation from bone marrow progenitor cells. The human use experience of Eltrombopag in CLD patients followed its utilization in chronic refractory ITP population [28]. Its efficacy and safety for the treatment of thrombocytopenia in patients with CLD was first evaluated in large randomized, double-blind, placebo-controlled trials, Eltrombopag to Initiate and Maintain Interferon Antiviral Treatment to Benefit Subjects with Hepatitis C-Related Liver Disease (ENABLE 1 and ENABLE 2), where it was used to increase platelet counts in thrombocytopenic patients prior to HCV treatment with interferon based therapies [29]. The use of Eltrombopag increased platelet numbers in thrombocytopenic patients with HCV and advanced fibrosis and cirrhosis, thus allowed otherwise ineligible or marginal patients to begin and maintain antiviral therapy, and led to significantly increased rates of HCV cure. The median time to achieve the target platelet count was approximately 2 weeks and 95% percent of patients were able to initiate antiviral therapy. The majority of patients treated with Eltrombopag (76%) maintained a platelet count greater than or equal to 50,000/*μ*L compared with 19% for placebo. Also a greater proportion of patients on Eltrombopag did not require any antiviral dose reduction as compared with placebo (45% versus 27%) [29]. The differences in SVR could be attributed to greater peginterferon exposure in the Eltrombopag arm as study investigators were required to lower peginterferon doses according to the product labels rather than clinical judgment. However, the study confirmed that Eltrombopag has potent platelet stimulatory effects in humans with liver disease.

Romiplostim is a recombinant fusion TPO mimetic protein that is administered intravenously or subcutaneously and leads to a dose-dependent increase in the platelet count. Through binding and activation of the TPO receptor, it activates intracellular transcriptional pathways, leading to increased platelet production. Majority experience of Romiplostim comes from clinical studies in chronic ITP population [30]. Only anecdotal case reports are available regarding its utility in CLD patients with thrombocytopenia [31].

Avatrombopag is an orally administered, small nonpeptide TPO receptor agonist that is shown to mimic the biological effects of TPO both in vitro and in vivo [32]. It is being utilized in clinical trials including patients with chronic ITP [33] and in CLD (NCT01972529) [34].

### 3.1. Eltrombopag

Eltrombopag was shown to safely increase platelet counts in patients with thrombocytopenia associated with cirrhosis due to chronic HCV infection in a phase II study [35]. Afdhal et al. [24] conducted an international, phase III, double-blinded, randomized, placebo-controlled clinical trial (Eltrombopag Evaluated for Its Ability to Overcome Thrombocytopenia and Enable Procedures, ELEVATE) that assessed the utility of Eltrombopag to increase platelet counts and reduce the need for platelet transfusions in patients with thrombocytopenia and CLD who were undergoing an elective invasive procedure. 292 adult subjects were enrolled between June 2008 and September 2009 and, among those, 252 (86%) had clinically or biopsy proven cirrhosis (10% had Child-Pugh C with score of 10–12 and Model for End-stage Liver Disease, MELD, score ≤ 24) with a platelet count of less than 50,000/*μ*L. All of the subjects otherwise needed a platelet transfusion before the procedure, according to local guidelines at each site. Exclusion criteria included pregnancy, abdominal imaging evidence of Portal Venous Thrombosis (PVT) within the 3 months prior to enrollment, presence of risk factors or a prior history of arterial or venous thrombosis, any condition associated with World Health Organization (WHO) grade 3 or 4 bleeding, or an active infection. The subjects were randomized 1 : 1 to receive either Eltrombopag (75 mg orally daily) or a placebo for 14 days and platelet counts were measured on day 8 and day 15. Invasive procedures were scheduled within 5 days after the patient received the last dose of Eltrombopag. On the day of the procedure, patients with a platelet count of more than 80,000/*μ*L did not receive a platelet transfusion and those with a platelet count of less than 50,000/*μ*L received a platelet transfusion before the procedure. The subjects with platelet counts between 50,000/*μ*L and 80,000/*μ*L received transfusion per the protocol at their respective centers. The primary end point was the number of subjects who did not require a platelet transfusion prior to, during, and up to 7 days after the procedure. On day 1 of the study, 94% of subjects in the placebo group and 92% of those receiving Eltrombopag had platelet counts of less than 50,000/*μ*L. On day 15, 59% of the subjects treated with Eltrombopag (compared with 5% in the placebo group) had a platelet count of more than 80,000/*μ*L. The majority of the subjects in both arms underwent various invasive procedures with similar bleeding risk profiles. The primary end point (avoidance of platelet transfusions) was achieved in 72% (104/145) of the subjects who received Eltrombopag, compared with 19% (28/147) in the placebo group (*p* < 0.001). The most common adverse effects were headache, pyrexia, and abdominal pain, which were observed equally in the treatment and placebo groups. Eight subjects had 10 thrombotic events: 6 subjects from the treatment group and 2 subjects from the placebo group. Nine of the 10 events involved the portal venous system (PVT) and none of the events occurred during the therapy. Of the 6 subjects in the Eltrombopag group who had PVT, 5 had a platelet count higher than 200,000/*μ*L. No significant difference was observed between the treatment (17%) and placebo (23%) groups in terms of bleeding episodes of WHO grade 2 or higher. The median number of platelets units administered to patients during each transfusion episode was lower in the Eltrombopag group compared with the placebo group (3.0 versus 4.0, resp.). Rates of bleeding episodes were nonsignificant for noninferiority (23% in the placebo group and 17% in the study group). Thus, the daily use of 75 mg of oral Eltrombopag for 14 days raised platelet counts and appreciably reduced the proportion of patients requiring a platelet transfusion prior to elective procedure. Analyses of secondary efficacy end points showed that fewer platelet units were transfused in the Eltrombopag group than in the placebo group and that the bleeding episodes observed in the Eltrombopag group were statistically noninferior to those in the placebo. Of note, the practice with regard to the use of platelet transfusions for thrombocytopenia was not standardized across the centers in this study for those with preprocedure platelet counts less than 80,000/*μ*L. In total, 20% of CLD patients with thrombocytopenia had WHO grade 2 or higher degree of spotted bleeding, and Eltrombopag did not reduce the events despite improving the platelet numbers. About 4% of the patients who received Eltrombopag were discovered to have PVT. The authors theorized that the sustained increase in platelet count and the predisposing injury from the procedure contributed to the development of the thrombosis. However, it should be noted that Doppler ultrasonography of the abdomen was not a prerequisite during the screening period, so it can be expected that some of the patients at study entry had a subclinical partial PVT or a low flow state, which could contribute to the development or persistence of thrombosis during the study. In the post hoc analysis, an increase in the platelet count to 200,000/*μ*L or higher was associated with an increased risk of thrombotic events [24]. In clinical practice, a use of different dosing regimen (a decreased dose, less-frequent dosing, or a shorter duration of dosing) could minimize the proportion of patients who have a platelet count of 200,000/*μ*L or higher and thus reduce the risk of PVT with Eltrombopag.

### 3.2. Romiplostim

 Moussa and Mowafy [25] conducted single arm, open label study to evaluate the efficacy of Romiplostim in subjects with HCV cirrhosis and thrombocytopenia who had failed to respond to standard treatment to correct thrombocytopenia prior to an elective procedure by enrolling patients from March 2009 to March 2010 in a single center. All of the 35 included subjects had documented chronic HCV with cirrhosis, classified as Child-Pugh score C, with platelet counts of less than 50,000/*μ*L, and had failed standard treatments (such as platelet transfusion, antioxidants, and/or folic acid). All of the subjects had a planned nonemergent surgical procedure scheduled. Those with the presence of bone marrow fibrosis, acute leukemia, myelodysplasia, or a history of thromboembolic conditions were excluded from the study. All subjects received a fixed dose of 2 *μ*g/kg subcutaneously (SC) every week for a maximum of 4 weeks or until 2 consecutive platelet counts of above 70,000/*μ*L were observed. The primary end point of the study was defined as the number of subjects achieving a platelet count of more than 70,000/*μ*L; the platelet count was assessed on day 0 and every 3 days for 90 days. A rapid response to Romiplostim therapy was observed, and 33/35 subjects achieved platelet counts above 70,000/*μ*L (mostly between day 12 and day 18) and, thus, underwent surgery without any bleeding complication. Seven of the 35 subjects experienced positive long-term effects of the Romiplostim, defined as a platelet count remaining above 50,000/*μ*L after the end of the platelet count peak (day 42). Although no serious adverse events were observed in this study, headaches were reported among patients receiving the placebo and the study drug. None of the patients had a thromboembolic event, which was evaluated by sonographic and portal duplex imaging. Importantly, none of the patients had a bleeding episode postoperatively. Thus, it was shown that treatment with Romiplostim for correction of severe thrombocytopenia in patients with liver cirrhosis resulted in an increase in the platelet count to a level needed for these patients; thus, they were able to undergo a nonemergent procedure without any significant complications [25]. The Romiplostim product label recommends an initial dose of 1 *μ*g/kg body weight, increasing by increments of 1 *μ*g/kg to a maximum of 10 *μ*g/kg, and indicates vigilant use in patients with CLD, in the presence of platelet counts above the normal range or other risk factors for thromboembolic events. A more conservative treatment dosing regimen was administered in this present study and, thus, no serious adverse events were reported without any episode of postoperative bleeding. In addition, none of the patients experienced a thrombotic event. More than 90% of the patients achieved a suitable platelet count for them to undergo their elective surgical procedure. In addition, it was shown that the use of Romiplostim cost about $500–600 less when compared to the cost of platelet transfusions [25].

### 3.3. Romiplostim versus Eltrombopag versus Platelet Transfusion

Basu et al. [26] conducted a single center, prospective, randomized, double-blind study in which 65 subjects with CLD and thrombocytopenia (baseline platelet count of <60,000/*μ*L) received a TPO agonist or platelets transfusion prior to undergoing a percutaneous liver biopsy. The study evaluated the single use of Romiplostim two weeks prior to a liver biopsy procedure as compared with Eltrombopag and platelet transfusions per center protocol. All of the subjects in this study had liver cirrhosis (mean MELD score 20) secondary to HCV (57%), HBV (15%), NASH, and alcoholic or primary biliary cirrhosis. The exclusion criteria were as follows: patients with ITP, drug induced thrombocytopenia, HIV, HCC, hemangiomas, autoimmune thrombocytopenia, use of steroids, and/or myelodysplastic syndrome. The randomization was performed as follows: Group A with 18 subjects to receive 7 pools of platelets prior to the night prior to the procedure, Group B with 23 subjects to receive a single dose of SC Romiplostim 500 mcg two weeks prior to the procedure, and Group C with 24 subjects to receive oral Eltrombopag 75 mg daily each day for 2 weeks prior to the procedure. All of the subjects had a baseline mean platelet count of less than 60,000/*μ*L prior to enrollment. A platelet count was repeated the day of biopsy and 4 weeks after the procedure. In Group A, the prebiopsy platelet count after transfusions was 183,800/*μ*L and returned to baseline at 4 weeks after the biopsy. In Group B, the prebiopsy platelet count rose to 232,000/*μ*L (significantly higher than Group A and Group C) and stayed significantly elevated 4 weeks after procedure at 366,200/*μ*L (*p* < 0.001 versus Group A and Group C). In Group C, the prebiopsy platelet count was increased to 189,900/*μ*L (no statistical difference compared to Group A); however, 4 weeks after procedure, it was significantly higher at 173,600/*μ*L compared to Group A only. The single dose of Romiplostim was the least expensive method, costing $2284, compared to $7500 for the platelet transfusion and $2991 for the Eltrombopag administration. The adverse effects observed in this study were postinjection site erythema (39%), arthralgia (15%), headache (13%), and/or nausea (8.8%). In addition, no postbiopsy bleeding or hematoma was noted. Although occurrence of PVT, hepatic decompensation, and death was not available in the preliminary study report, the results suggested that the single use of 500 mcg of SC Romiplostim was efficacious, cost-effective, and safe with minimal adverse effects when compared to platelet transfusions and Eltrombopag in patients with cirrhosis and thrombocytopenia needing a liver biopsy.

### 3.4. Avatrombopag

Terrault et al. [27] conducted an international, phase II, randomized placebo-controlled, double-blind trial to evaluate the utility of Avatrombopag in correcting thrombocytopenia in 130 cirrhotic subjects prior to an elective procedure. The included subjects with chronic liver disease were mainly from viral hepatitis (80%), NASH, or alcoholic liver disease; and their MELD scores were less than 24. All of the subjects had clinically proven cirrhosis and 13% of the included subjects had Child-Pugh C disease. All had 2 independent baseline platelet counts ranging from 10 to 58,000/*μ*L and underwent an elective procedure 1 to 4 days after the completion of the dosing schedule of Avatrombopag or the placebo. Those with primary hematologic disorder, ITP of any cause, and/or a history of arterial or venous thrombosis were excluded. Subjects were scheduled to undergo a wide variety of procedures including colonoscopy with or without polypectomy, esophagogastroduodenoscopy with or without banding, bronchoscopy, dental procedure, transarterial chemoembolization of HCC, right heart catheterization, radiofrequency ablation of HCC, liver biopsy, paracentesis, TIPS placement, and hernia repair. Subjects were randomized to two control arms along with Avatrombopag in two intervention cohorts as Cohort A (4 arms) who received either a placebo or 1 of 3 different doses of first-generation oral Avatrombopag formulation (100 mg loading dose followed by 20, 40, or 80 mg/day on days 2–7) and Cohort B (3 arms) who received either a placebo or 1 of 2 different doses of second-generation oral Avatrombopag formulation (80 mg loading dose followed by 10 mg/day for days 2–7 or 20 mg/day for days 2–4). In phase I studies, the second-generation formulation produced about 1.6 times the exposure relative to the first-generation formulation. Thus, dosing was adjusted for study arms. Study's primary end point was defined as an increase in platelet count by 20,000/*μ*L above baseline and at least one platelet count of above 50,000/*μ*L from days 4 to 8. This was achieved by 49% of the treated subjects in Cohort A (compared to 6.3% in the control arm) and 47.6% in Cohort B (compared to 9.5% in the control arm). Excluding the 100/40 mg arm in Cohort A (*p* = 0.17), each of the Avatrombopag subcohorts had a high percentage of responders (subjects achieving a platelet count >75,000/*μ*L or >100,000/*μ*L at least once within days 4–8) compared with their respective placebo arm (*p* < 0.01). Nausea, fatigue, and headache were among the most common adverse effects, which were noted in the treatment and placebo group. Serious adverse events occurred in 10.8% of placebo and 17.9% of Avatrombopag treated group (*p* = 0.36); most of these events were complications of underlying cirrhosis, such as ascites and hepatic encephalopathy. Hepatic decompensation constituted the main serious adverse effect and was similar in combined study (17.9%) and placebo (10.8%) groups (*p* = 0.36) [27]. There was 1 death after the planned procedure in Cohort B (80/10 mg arm); the death was thought to be possibly related to the study drug, but the patient also had preexisting cardiopulmonary disease. A single case of PVT was identified in Cohort A (100/80 mg arm) that was diagnosed on study day 34. This PVT was then successfully managed with a portal vein thrombectomy, followed by warfarin anticoagulation. Bleeding episodes as a procedure related adverse event were not assessed in this study; however, only 4 episodes of GI bleeding were observed. Thus, this study showed that the use of Avatrombopag achieved significant increases in platelet counts 3–7 days after treatment in approximately 50% of subjects and in up to 75% of those receiving the highest dose, thus suggesting a potential treatment for thrombocytopenia in patients with advanced CLD undergoing elective invasive procedures in a week's time. The dose-dependent rise in platelet count prior to procedure was not evaluated as a marker of reduced risk of bleeding. This study started off using the first-generation formulation of Avatrombopag and subsequently included a separate cohort and dosing regimen to use second-generation formulation of this TPO agent (thus completed phase II analysis for the ongoing phase III study with second-generation Avatrombopag). The patient who developed a PVT had peak platelet count reaching 199,000/*μ*L, with a platelet count at time of diagnosis of 55,000/*μ*L and Doppler sonography scan performed three months prior to study entry demonstrated evidence of a very low portal vein flow. This study used Doppler scans and/or MRI and CT imaging only in Cohort B and thus removed patients with PVT at screening. Of note, platelet transfusion was not standardized or evaluated in this study as an end point [27]. Phase III studies will utilize Avatrombopag to evaluate the rate of platelet transfusion as a primary end point in order to confirm and extend the finding of correction of thrombocytopenia prior to the procedures in cirrhosis patients.

## 4. Conclusion

Thrombocytopenia is frequently present in the patients with CLD who require an invasive procedure as part of their routine clinical care. In the study evaluating the bleeding risk among patients with severe thrombocytopenia (platelet count less than 75,000/*μ*L), it was found that 31% of the patients had bleeding complication related to procedure [36]. Platelet transfusions are often used to increase patient's platelet count—to reduce the anticipated risk of bleeding, in either the setting prior to a procedure or an elective surgery. In addition, platelet transfusions were routinely done prior to initiation of interferon therapy in patients with HCV and thrombocytopenia to minimize the dose adjustments or therapy interruptions. Platelet transfusions have many drawbacks, including transfusion reactions, short efficacy, and the development of refractory thrombocytopenia due to the development of anti-platelet antibodies from receiving multiple transfusions. The use of TPO agonists seems to have merits of avoiding transfusion related complications and have shown relative efficacy in improving platelet counts in CLD in various clinical scenarios [29, 35]. To date, limited data is available for studying the use of such agents to improve platelet number and thus reduce the bleeding complications related to invasive procedures in CLD patients.

Analysis of large phase III ELEVATE trial [24] delineated the need for further experience with the use of Eltrombopag therapy and assessment of risk factors for development of PVT for careful patient selection. Until further studies are performed, the clinical use of Eltrombopag is not recommended in CLD patients with thrombocytopenia prior to an elective invasive procedure. Based on the evidence provided by Moussa and Mowafy [25] with regard to Romiplostim therapy, further larger studies with a longer follow-up period are needed to define an optimal dosing schedule. More durable clinical outcome data is needed prior to the utilization of this agent in clinical practice. Further, Basu et al. [26] showed that single Romiplostim was efficacious, cost-effective, and safe with minimal adverse effects when compared to platelet transfusions and Eltrombopag in cirrhotic patients undergoing a liver biopsy with platelets less than 60,000/*μ*L. However, the data regarding mortality, hepatic decompensation, and thrombotic events was missing from this preliminary report on this study. The most recent clinical trial completed by Terrault et al. [27] elucidated that oral Avatrombopag increased platelet counts in a generally dose-dependent manner in patients with cirrhosis and thrombocytopenia; however, this effect was not assessed in relation to platelet transfusions or reduction in bleeding risks. At least 2 clinical trials are currently underway which will evaluate the efficacy of Avatrombopag in reducing the proportion of participants who require platelet transfusions or as a rescue therapy to reduce the bleeding risk before and after an elective procedure in patients with liver cirrhosis.

## Figures and Tables

**Table 1:** Studies evaluating TPO agonists in CLD patients.

Study medication (route of administration)	Dosing regimen	Study population	Procedures performed	Primary outcome	Secondary outcomes	Adverse effects
Eltrombopag (oral) Afdhal et al. [24]	75 mg/day for 2 weeks versus placebo for 2 weeks prior to procedure	*N* = 292 subjects with CLD and platelet count of <50,000/*μ*L and a planned elective procedure Cirrhosis: 86% (10% Child-Pugh C) Etiology: 80% viral hepatitis	Majority: 59% low bleeding risk procedures (endoscopy, interventions, and paracentesis) Also 15% mild bleeding risk procedures (liver biopsy, laparoscopy, and HCC ablation)	Subjects in whom a platelet transfusion was avoided before, during, and up to 7 days after the elective invasive procedure: 72% (104/145) in the study group compared with 19% (28/147) in the placebo group did not require platelet transfusion (*p* < 0.001)	Rate of bleeding episodes: nonsignificant for noninferiority (23% in the placebo group and 17% in the study group) Quantity of platelets units transfused: median of 3 units in the study group was lower than 4 units in placebo group Side effects: similar in both groups (headache, pyrexia, and abdominal pain, being most common)	Deaths: 3 in study group (with 1 due to sepsis and possibly related to therapy) versus 2 in placebo group Hepatic decompensation: similar in both groups, most common being hepatic encephalopathy PVT: 9 events identified (7 in study group versus 2 in placebo, OR 3.04)

Romiplostim (SC) Moussa and Mowafy [25]	2 *μ*g/kg SC every 1 week for a maximum of 4 weeks or until 2 consecutive platelet counts of >70,000/*μ*L were observed prior to procedure	*N* = 35 subjects with refractory thrombocytopenia and platelet count of <50,000/*μ*L and a planned surgical procedure Cirrhosis: 100% (all with Child-Pugh score of 10 and above) Etiology: HCV	Cataract, hernia repair, joint replacement, and fracture fixation	The number of subjects achieving a platelet count >70,000/*μ*L and thereby eligible for surgery: 94% achieved this end point and underwent procedure	Platelet response: 33/35 achieved the desired goal in 9–30 days. All subjects showed rapid response and 7/35 showed positive long-term response (a platelet count > 50,000/*μ*L at 3-month follow-up) Safety: no serious adverse effects reported	Deaths: none Bleeding episodes: none Hepatic decompensation: none PVT: none identified

Romiplostim (SC) versus Eltrombopag (oral) versus platelet transfusion Basu et al. [26]	500 mcg SC (as a 1 time dose) given 2 weeks prior to the procedure versus 75 mg/day for 2 weeks till the day of procedure versus 7 units of platelet transfused the night prior to procedure	*N* = 65 subjects with baseline platelet count of <60,000/*μ*L prior to study Cirrhosis: 100% (mean MELD score 20) Etiology: 57% HCV, 15% HBV, and other diseases	Outpatient percutaneous liver biopsy	Platelet response: Romiplostim group had significantly higher prebiopsy and postbiopsy platelet counts as compared to the other groups	Cost effectiveness: Romiplostim single dose is cost-effective approach Adverse effects: injection site erythema (39%) being most common	Death: not available Postbiopsy bleeding: none Hepatic decompensation: not available PVT: not available

Avatrombopag: first-generation and second-generation formulation (oral) Terrault et al. [27]	Cohort A (first-generation Avatrombopag group): 100 mg loading dose followed by 20, 40, or 80 mg/day on days 2–7 in three parallel arms and also Cohort B (second-generation Avatrombopag group): 80 mg loading dose followed by 10 mg/day for days 2–7 or 20 mg/day for days 2–4 in two parallel arms versus placebo with each study cohort	*N* = 130 subjects with CLD and thrombocytopenia (baseline platelet count of <50,000/*μ*L) who were scheduled to undergo elective procedure Cirrhosis: 100% (13% Child-Pugh class C) Etiology: 80% viral hepatitis	Majority had endoscopic procedures Also dental procedure, liver biopsy, and paracentesis	An increase in platelet count >20,000/*μ*L above baseline and at least one platelet count >50,000/*μ*L within days 4–8 was achieved by 49% of treated subjects in Cohort A and 47.6% in Cohort B compared to 6.3% and 9.5% in placebo The proportion of subjects achieving a platelet count >75,000/*μ*L or >100,000/*μ*L at least once within days 4–8: each Avatrombopag regimen had a higher proportion of responders compared with their respective cohort placebo arms (*p* < 0.01)	Platelets response: the maximum median platelet count increase from baseline in all study subjects occurred within 10–13 days. A platelet count >100,000/*μ*L prior to procedure occurred in 17.6% in subjects with Avatrombopag and none in placebo group Safety: overall similar rate of adverse events in combined study (29%) and combined placebo group (29.7%)	Death: 1 in study (80/10 mg) arm possibly related to study drug Hepatic decompensation: constituted mainly serious adverse effects and were similar in combined study (17.9%) and placebo (10.8%) groups (*p* = 0.36) Bleeding episodes: only 4 subjects had gastrointestinal bleeding episodes PVT: 1 in 100/80 mg arm

## Additional Points

*Key Messages*. (1) Severe thrombocytopenia is a frequent hemostatic abnormality seen in chronic liver disease patients and is associated with increased bleeding risk with invasive procedures. (2) Impaired production of thrombopoietin is considered to contribute to the development of thrombocytopenia in chronic liver disease. (3) Platelet transfusions, splenic artery embolization, splenectomy, and Transjugular Intrahepatic Portosystemic Stent placement are invasive methods to correct thrombocytopenia in chronic liver disease patients. (4) Initial studies have shown thrombopoietin receptor agonists to be beneficial in these patients especially to improve platelet counts prior to elective invasive procedures. More studies are needed to evaluate the efficacy of thrombopoietin receptor agonists to correct thrombocytopenia in chronic liver disease patients, specifically in regard to the reduction in bleeding risks related to invasive procedures and minimizing the complications (such as portal vein thrombosis) among different agents.
